# Nitrate of Silver as a Local Remedy in Phlegmasia Dolens

**Published:** 1855-04

**Authors:** J. R. Mills

**Affiliations:** Huntington, Ind.


ART. II.—*Nitrate of Silver as a local remedy in Phlegmasia Dolens.* By J. R. MILLS, M.D., of Huntington, Ind.

MESSRS. EDITORS.—I saw in your Journal some time since (if I am not mistaken) the report of a case where some gentleman M.D. had used the nitrate of silver in Phlegmasia Dolens successfully. The idea struck me as a good one, and I determined to give it trial the first opportunity, and that happened recently in a case of M. Y., aged 30, and in her fourth confinement. Dr. W. was her attending physician When I was called in consultation he informed me that the leg had commenced swelling on the fifth day after her confinement, and had continued to do so until I saw her, which was on the 12th day. I recommended the painting of the leg entire with a strong solution of the nitrate of silver of sufficient strength to produce vesication after the second application. Upon opening the blister the fluid seemed of an opaque color, and of more consistence than in common cases. I directed it to be dressed with cabbage leaves for four days, by which time the pain had entirely subsided, the swelling nearly gone, and the patient could flex or extend the leg at will without much inconvenience. I think the remedy deserves further trial from the profession.

